# From detection to action: artificial intelligence in integrated pest and invasive plant management

**DOI:** 10.1007/s44307-026-00118-7

**Published:** 2026-07-08

**Authors:** Yaoxing Li, Lianming Zha, Weitong Liu, Feng Luo, Chenyang Xu

**Affiliations:** School of Agriculture and Biotechnology, Shenzhen Campus of Sun Yat-Sen University, Shenzhen, 518107 China

**Keywords:** Precision Agriculture, Integrated Pest Management, Artificial Intelligence

## Abstract

Global agriculture faces growing threats from pests, pathogens, and invasive species, intensified by climate change and biodiversity losses. Conventional approaches are limited in both precision and scale, and artificial intelligence (AI) is now reshaping integrated pest management (IPM). Modern agricultural monitoring leverages high-resolution observations, hyperspectral sensors, and the Internet of Things (IoT) to facilitate early detection for diseases. Hybrid AI systems can integrate multi-source data to enhance the accuracy of real-time monitoring of pest and disease dynamics, including the detection and tracking of sparse invasive populations and their biomass even under shifting climate scenarios. They further enable predictive forecasting and the optimization of management strategies. When AI-driven diagnostics are integrated with autonomous robotics, they form a robust framework for epidemic mitigation. Here, we provide a systematic macro-perspective review, bridging foundational AI mechanisms with actionable IPM intelligence. We analyze the evolution of agricultural AI from cross-modal architectures to multi-scale diagnostics and full-lifecycle interventions. Finally, we propose a framework for the global agroecological network, offering a sustainable path toward maximizing productivity while ensuring ecological resilience.

## Introduction

Vegetation plays a foundational role in maintaining terrestrial ecosystems, ensuring global food security, preserving biodiversity, and regulating climate dynamics (Li et al. 2024a; Muluneh 2021). While forests, grasslands, and agricultural biomes provide critical ecosystem services, they are increasingly threatened by pests, pathogens, and invasive species. Accelerated by climate change, biological disturbances have become more frequent and widespread, threatening the stability of both global agriculture and natural habitats (Carrasco et al. 2010; Deutsch et al. 2018; Prasad et al. 2010; Skendžić et al. 2021). The scale and complexity of these biological threats have exposed fundamental weaknesses in traditional surveillance frameworks. Conventional monitoring methods, constrained by labor-intensive manual inspections, subjective visual assessments, and reactive workflows, becomes increasingly inadequate to catch the speed of modern epidemics (Barbedo 2016; Esfandi et al. 2024). Currently, plant health management is undergoing a paradigm shift, transitioning from experience-based methods toward high-dimensional, data-driven inference. The integration of Artificial Intelligence (AI), remote sensing, and robotics into Integrated Pest Management (IPM) constitutes a structural evolution rather than a mere technological upgrade. This transition enables earlier detection, higher diagnostic precision, and targeted interventions.

AI applications have advanced from simple image-classification tasks to integrated systems capable of combining physical process information with biochemical trait inference. This trajectory is fueled by breakthroughs in deep representation learning, cross-modal data fusion, and edge intelligence, supported by IoT networks, hyperspectral imaging, and autonomous aerial systems. Despite these advancements, significant barriers remain for real-world deployment. Environmental heterogeneity, limited computing infrastructure in rural areas, and a lack of standardized intervention protocols retain the gap between experimental success and field applicability (Miller et al. 2025; Sun et al. 2026). Furthermore, biological invasions present a unique challenge: the extreme phenotypic plasticity of invasive species, combined with a chronic scarcity of annotated training data, severely hinders the development of robust early-warning models (Chowdhury et al. 2024; Gruntman et al. 2020). As climate-driven ecosystem disruptions accelerate, it becomes even more imperative to safeguard global vegetation. The convergence of AI and IPM signifies a fundamental paradigm shift toward precision agriculture and conservation. In this review, we systematically align methodological innovations with specific operational requirements and agricultural imperatives. This integration offers a transformative pathway to harmonize productivity with ecological stewardship, fostering the long-term resilience of global vegetation. As environmental pressures continue to mount, these intelligent systems offer a pragmatic and robust framework for building sustainable agroecological networks.

**Fig. 1:**
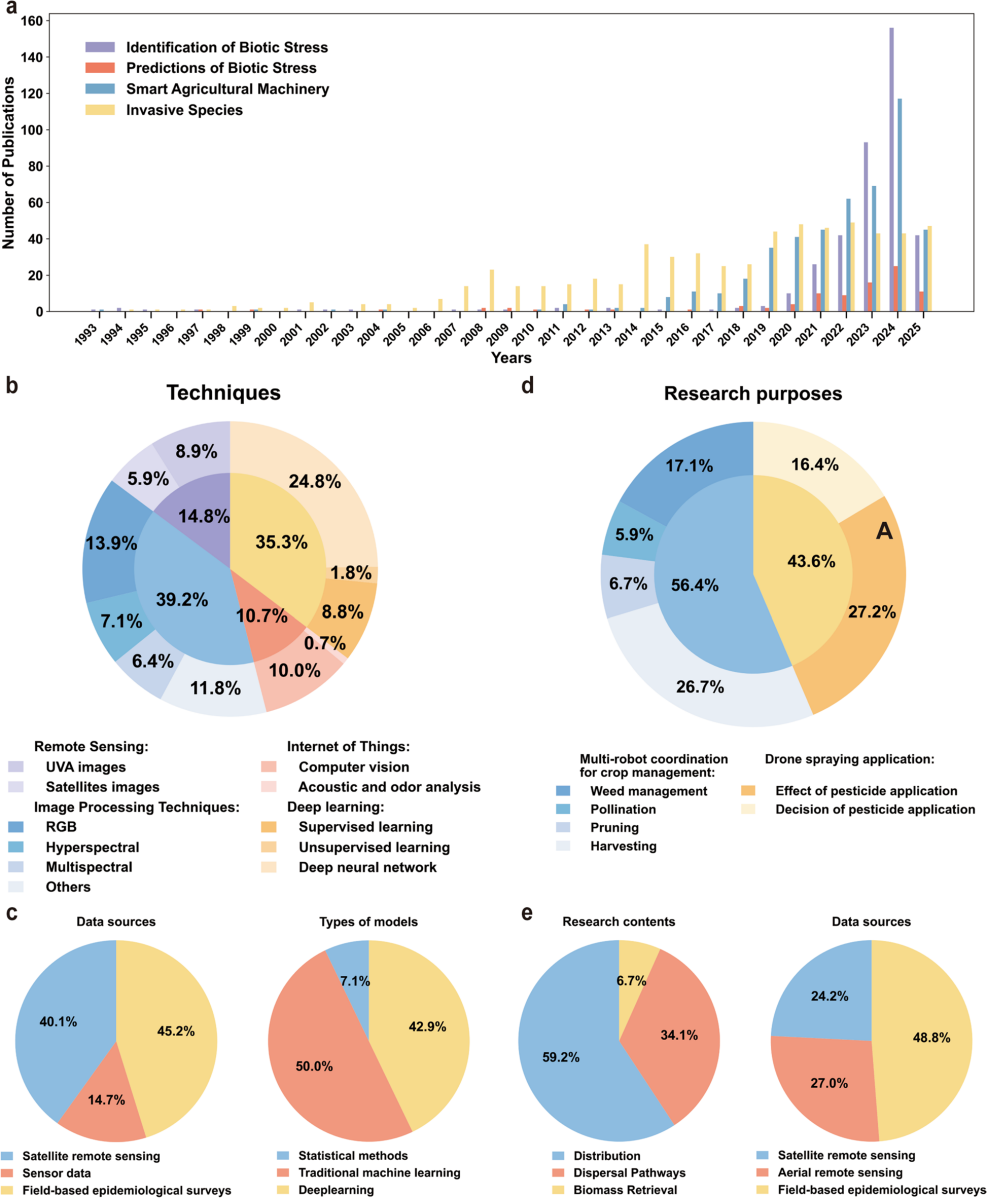
The application of AI technology from 1990s-2020s. **a** Number of published papers about the application of AI technology in identification of biotic stress, predictions of biotic stress, intelligent agricultural machinery and invasive species. **b** Percentage of technical classifications in identification of biotic stress. **c** Percentage of different data sources and types of models in predictions of biotic stress. **d** Percentage of different research purposes in intelligent agricultural machinery. **e** Percentage of different research contents and data source in invasive organisms

We conducted a systematic review of literature in Scopus, Web of Science, and IEEE Xplore, spanning 1993 to 2025. Our analysis identifies distinct temporal and thematic patterns in the application of intelligent technologies across four key dimensions. Before 2010, applications of AI were limited by computational and data constraints, whereas research on invasive species detection grew steadily as their ecological and economic impacts became better understood. After 2010, breakthroughs in machine learning spared a surge in research on automated identification and intelligent machinery. Despite this growth, significant imbalances remain. Predictive modeling lags substantially behind identification (Fig. 1A). While region-based deep learning (DL) has become the dominant architecture for high-precision identification (Fig. 1B), predictive tasks still rely heavily on traditional statistics, leaving the potential of DL largely underutilized (Fig. 1C). Furthermore, the surge in drone-based spraying systems over the last decade underscores their rising importance in precision agriculture (Fig. 1D). Meanwhile, although the distribution and dispersal pathways of invasive species are well documented, biomass estimation is still underexplored and requires urgent focus (Fig. 1E).

**Fig. 2:**
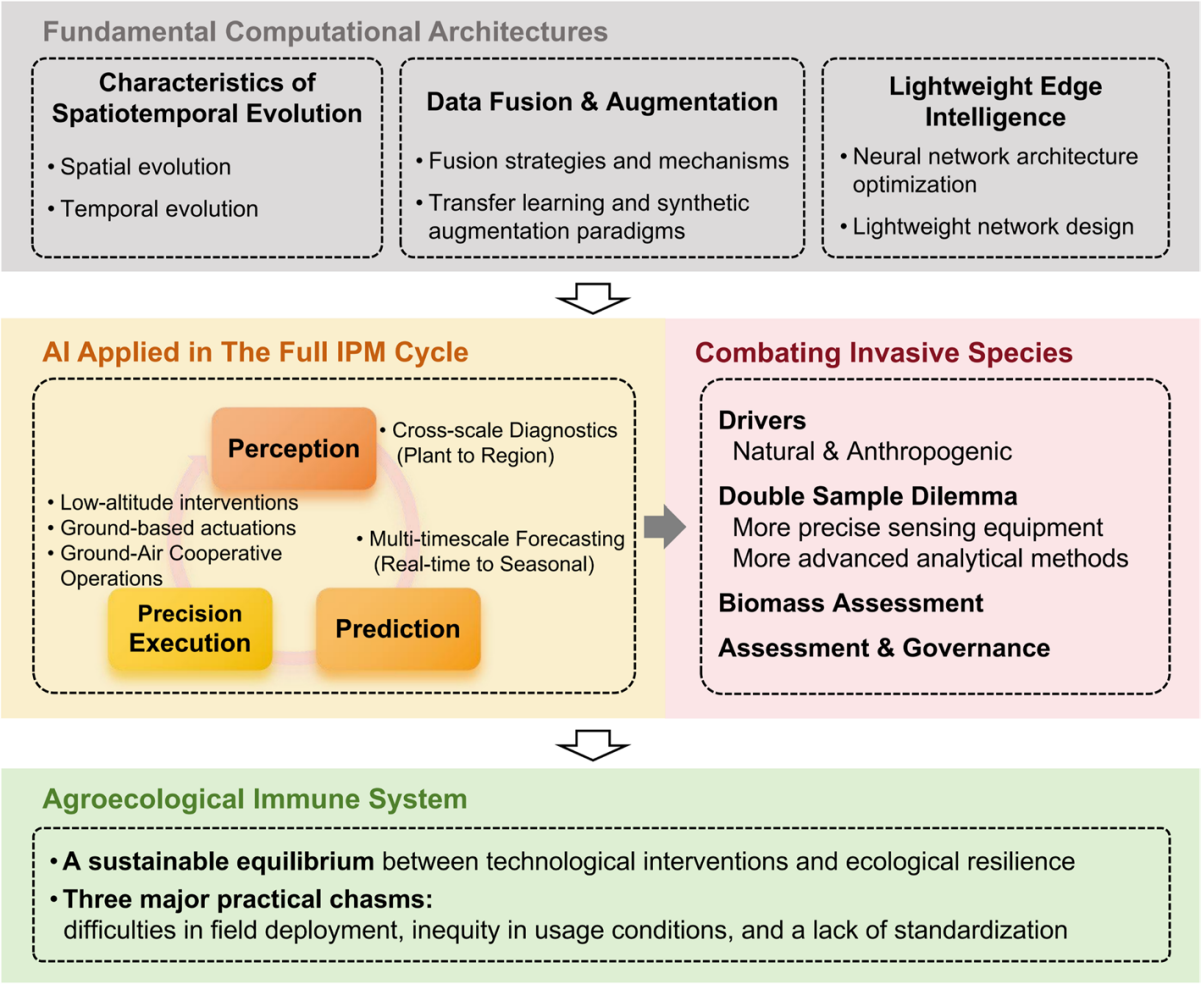
Framework of AI application in integrated pest management

This review synthesizes existing studies to provide a systematic roadmap for both phytopathologists and computational scientists. The core objective is to elucidate how AI mechanisms can be translated into actionable IPM strategies (Fig. 2). The review is organized into four core modules: 1) An analysis of the architectures supporting agricultural intelligence, including multidimensional feature extraction and cross-modal synergy. 2) A detailed examination of the algorithms in practice, ranging from precision diagnostics and climate-driven spatiotemporal forecasting to life-cycle-integrated interventions. 3) A specialized focus on ecological risk assessment and management under conditions of severe data scarcity. 4) A concluding synthesis of deployment considerations and a vision for a globally interconnected, AI-enabled agroecological immune network.

## AI-driven core technologies for IPM

### Spatiotemporal evolution of plant disease intelligence

The core of modern intelligent pest management lies in translating physiological plant stress and environmental disturbances into computable mathematical representations. This process has undergone a shift from expert-dependent feature engineering to data-driven latent feature learning. This section elucidates how underlying algorithms extract and reconstruct critical information across spatial and temporal dimensions.

#### Spatial evolution of plant disease recognition

Early disease recognition relied heavily on traditional image processing techniques where researchers manually defined the geometric shapes and textural patterns of leaf lesions to be matched against predefined libraries by basic classifiers (Min et al. 2025). However, these handcrafted approaches proved fragile in complex field environments characterized by shifting illumination and cluttered backgrounds. The advent of deep learning architectures reshaped these perception capabilities, as convolutional neural networks (CNNs) began extracting hierarchical features directly from pathological imagery. By utilizing multi-layered convolutional operations, these models eliminate the need for manual feature engineering and instead learn to recognize objects from low-level edge structures to high-level lesion textures, providing superior robustness in real-world visual environments (Zheng et al. 2018; Wadmare et al. 2024).

While standard RGB imaging is effective for extracting late-stage morphological features, its inherent physiological blind spots preclude the detection of pre-symptomatic stress. To achieve earlier detection, feature representation expands beyond the visible spectrum into non-visible wavelengths. Multispectral and hyperspectral imaging (HSI) capture unique reflectance signatures that reveal internal biochemical changes before physical symptoms become visible (Al-Saddik et al. 2018). Concurrently, thermal and chlorophyll fluorescence imaging allow for the quantification of physiological metabolic stress through indicators such as reduced fluorescence intensity. In processing high-dimensional data, advanced computational architectures enable the joint extraction of spatial and spectral features, effectively reducing inter-channel redundancy while capturing relationships across distant regions. This capacity for mining global spatial dependencies serves as the foundation for both broad field-scale perception and long-term spatiotemporal analysis.

#### Temporal evolution of plant disease analysis

Various biological stress signals exist as continuous data streams over time. Modern biosensors allow for the constant monitoring of these dynamic processes, capturing subtle shifts in sap flow or transpiration rates caused by infestations. Sequential models are used to process these dependencies, identifying specific physiological patterns that signal pest activity. This allows the system to distinguish between natural metabolic changes and the early signatures of biological stress.

Tracking the transnational spread of invasive species and forecasting climate trends requires analyzing data spanning several decades. While traditional models often struggle to maintain accuracy over such long periods (Saravana et al. 2026), Transformer-based architectures use attention mechanisms to process entire sequences at once (Guo et al. 2025; Santos et al. 2025). By identifying and prioritizing critical historical climate anomalies, these models can decode complex, large-scale patterns. This provides a robust foundation for predicting how invasive populations will move across diverse geographical regions over time.

### Heterogeneous fusion and data augmentation

In agroecosystems, illumination shifts, canopy occlusion, and extreme weather rapidly expose the limitations of single-sensor perception. To bridge these gaps, computational architectures rely on multi-modal fusion and cross-domain knowledge transfer.

#### Multi-source data fusion mechanisms

The core of modern agricultural AI is the integration of heterogeneous data across multiple dimensions. By aligning observable physical traits (from standard cameras) with internal biochemical changes (from spectral sensors), these systems significantly enhance diagnostic accuracy (Al-Saddik et al. 2018; Ispizua Yamati et al. 2025; Liu et al. 2025; Yu et al. 2025). Crucially, this multi-modal data fusion underpins effective air-ground synergy, bridging the spatial resolution gap between expansive aerial monitoring and localized ground-truth verification. For example, coarse data from satellites must be fused with high-resolution observations or ground-level imagery to create a complete picture of the field. At a global scale, this allows researchers to combine static land-cover data with dynamic climate sequences, forming high-resolution risk networks to track biological invasions.

#### Bridging data gaps through adaptive learning

DL typically requires various labeled data, which is often unavailable for newly emerged pests or rare invasive species. To overcome this, transfer learning leverages knowledge from related fields (Bondre and Patil 2023; Pandya and Jain 2023). This approach assumes that the basic logic for identifying shapes and textures is universal. By pre-training a model on large, general datasets and then fine-tuning it on a small number of specific agricultural samples, AI can be deployed rapidly in new scenarios with minimal data costs (Xu et al. 2022). When facing entirely novel threats, meta-learning takes this further. Instead of memorizing specific symptoms, the system learns a universal rule for adapting to new species traits (Pandey et al. 2022; Wu et al. 2023). In cases of extreme data scarcity, Generative Models, such as Generative Adversarial Networks (GANs), are used to synthesize realistic training data (Cho et al. 2024; Zhang et al. 2023c). By forging high-quality images of rare diseases or weather events, these models improve the system’s ability to recognize threats that have rarely been seen in the real world (Koumoutsou et al. 2022).

### Lightweight edge intelligence for in-situ operation

The advanced AI models used for deep feature extraction typically require high-performance, server-grade computing. However, IoT sensors and autonomous drones operate under strict power and hardware constraints. To enable real-time decision-making, agricultural AI shifts from centralized processing to lightweight, edge-oriented paradigms.

#### Neural network architecture optimization

The primary strategy for overcoming hardware barriers is model optimization through compression techniques such as pruning and quantization (Eccles et al. 2024; Gavel and Azad 2025; Gorvadiya et al. 2025). Pruning functions much like horticultural trimming, mathematically evaluates millions of connection weights and discards redundant neural pathways that contribute minimally to the final diagnosis. This significantly reduces the parameter count while maintaining performance (Gonzalez-Carabarin et al. 2021). Furthermore, quantization lowers the numerical precision of calculations to further decrease the memory footprint (Arcot et al. 2024; Liang et al. 2021). Together, these methods streamline monitoring models, enabling the deployment on low-power edge devices for rapid threat alerts.

#### Lightweight network design

Beyond shrinking existing models, architectures can be natively designed for edge hardware by decoupling complex operations to reduce redundancy. Traditional networks are computationally intensive because they process spatial and color data in a single, dense step. By mathematically factorizing these operations into sequential, simplified stages, computational costs are drastically cut while preserving the ability to extract relevant lesion features on low-power IoT devices (Nnamdi and Abolghasemi 2025). For tasks requiring immediate action, such as precision spraying, high-speed architectures resolve processing delays by analyzing an image in a single read rather than scanning it piece-by-piece (Qi et al. 2023). This one-stage approach simultaneously pinpoints a threat’s location and identifies the species, eliminating the need for repetitive calculations. These efficient designs are critical for the real-time, closed-loop control requirements in modern intelligent agricultural systems.

## Intelligent identification and diagnosis of diseases and pests

**Fig. 3:**
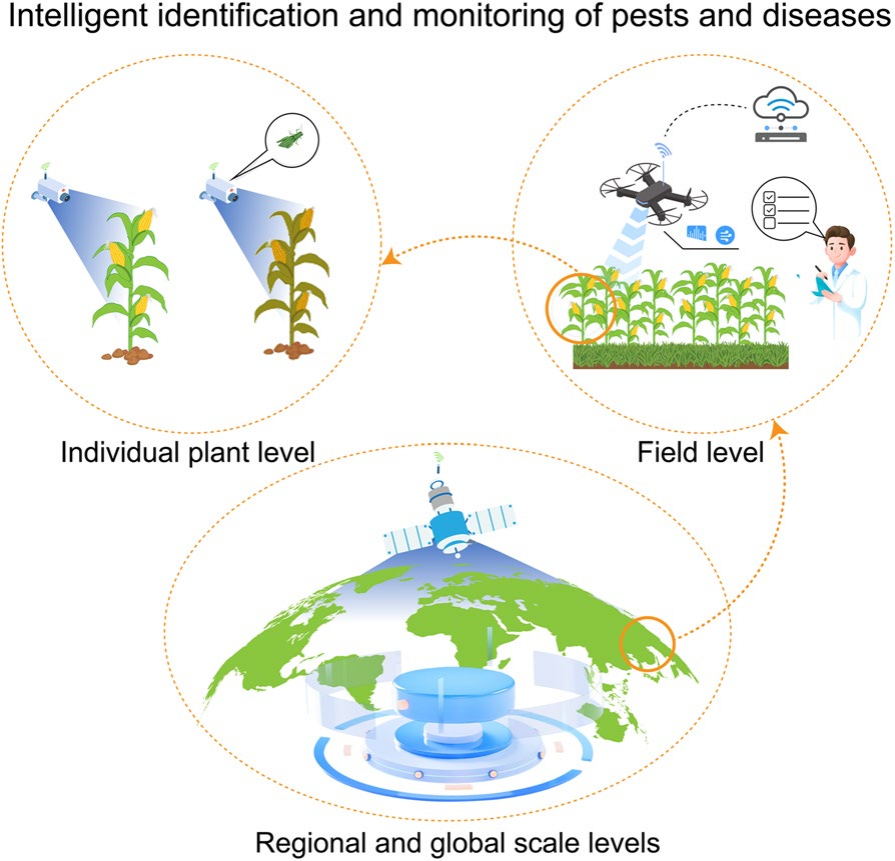
Multi-scale AI-enabled monitoring of pests and diseases in crops

Intelligent identification and monitoring of pests and diseases are mainly at three spatial scales: the individual plant level using close-range sensors, the field level using UAV-based imaging systems, and the regional to global levels via satellite-based remote sensing. These AI-integrated approaches enable timely and precise detection, enhancing responsiveness and management efficiency in IPM (Fig. 3).

### Micro-scale diagnostics for individual plants

Tools for plant phenotyping have become increasingly accessible, with standard cameras and portable spectrometers now widely used for organ-specific imaging. These advancements allow for high-accuracy pest and disease recognition by analyzing leaves, stems, and reproductive organs across the visible and infrared spectrums (Upadhyay et al. 2025; Yang 2010; Zhang et al. 2005).

#### Pre-symptomatic detection for latent threats

Early-stage infections often trigger internal physiological changes before symptoms are visible to the human eye. HSI is highly sensitive to these sub-visual shifts, enabling detection of viral latency in the ultra-early phase. To manage the high dimensionality of HSI data, machine learning is used to select the most relevant wavelengths, reducing computational complexity without losing diagnostic accuracy. The “spectral sensing & intelligent decision-making” framework decodes intricate biochemical correlations, providing a vital window for early intervention (Zhu et al. 2017; Zhang et al. 2024).

#### Post-symptomatic identification for visual pathological signatures

Once symptoms like leaf spots, eggs, or visible lesions appear, computer vision becomes the primary diagnostic tool. While traditional image processing is efficient for well-defined lesions in controlled settings, it struggles with complex patterns like pest camouflage or cluttered backgrounds. In contrast, DL models excel at learning these complex representations automatically. Hybrid architectures further improve performance by balancing precision and speed. For larger plants and trees, LiDAR is often employed to capture three-dimensional structural data. This technology accounts for the complex vertical layers of forest canopies, enabling an accurate assessment of defoliation and health even amidst environmental noise (Jiang et al. 2019; Li et al. 2021; Roy and Kukreja 2024).

### Meso-scale monitoring of pest and disease outbreaks

Field-level pest monitoring requires large-area coverage that cannot be achieved by individual plant-level tools. Field-scale analysis faces three key challenges: long-distance imaging limitations, multi-species discrimination under variable field conditions (Jing et al. 2018), and resilience to environmental interference like seasonal changes and lighting variations. Drones and IoT sensors track canopy traits and microclimate conditions in agricultural landscapes in real time (Al-Najadi et al. 2025), effectively addressing these challenges.

#### Navigating diverse canopy architectures

The success of drone-based monitoring depends heavily on the structure of the crop canopy. For broad-leaved crops with clear visual symptoms, drones can maintain high diagnostic accuracy even at standard altitudes. In denser or more complex canopies, flying at lower altitudes significantly improves the detection of specific diseases by capturing finer details (Calou et al. 2020; Garcia-Ruiz et al. 2013). For crops with smaller leaves or less distinct symptoms, a far-view and close-look strategy is employed. Drones first scan the entire field to identify anomalies, and then descend for high-resolution imaging of specific targets (Zhang et al. 2023b). This is complemented by ground-level IoT technologies and machine vision, which can quantify pest populations via video analysis. Together, these tools generate spatiotemporal heatmaps that track how pests and diseases move across a landscape over time (Jing et al. 2018).

#### Processing heterogeneous sensory signals

Beyond visual recognition, AI-driven IoT networks can detect invisible indicators of stress, such as physiological changes and chemical emissions (Li et al. 2024b). Some advanced sensors are bio-integrated, utilizing the plant’s internal energy to power long-term monitoring without requiring external power sources (XianYi et al. 2012). Acoustic sensors use specialized neural networks to identify the unique vibration signatures of wood-boring insects (Min et al. 2025). Similarly, electronic nose technologies monitor volatile organic compounds emitted by plants under stress, decoding gas signals to identify specific infections (Ren et al. 2025). Despite their potential, these methods face significant hurdles in open-field environments. Acoustic methods are limited to pests that produce distinct sounds, and chemical sensing is often disrupted by atmospheric interference and wind (Cellini et al. 2017; Miller et al. 2025). Transitioning these delicate laboratory-grade sensors into reliable, ruggedized field tools remains a key area for future research.

### Earth observation and macro-epidemiological monitoring

At regional and global scales, satellite remote sensing is indispensable for tracking pest and disease dynamics. Unlike ground-based methods, these systems provide continuous, multi-temporal observations over vast areas, allowing researchers to identify multi-year outbreak patterns (Zhang et al. 2019).

#### Large scale disease mapping and surveillance

In disease mapping and surveillance, the core task is to capture hidden signals, such as chlorophyll decline or water loss, across both spectral and temporal dimensions. Spectral features, particularly those utilizing red-edge bands, are exceptionally effective for early pathogen detection. Machine learning methods are employed to screen optimal bands from massive datasets, effectively eliminating redundant information and enhancing classification accuracy (Chen et al. 2018; Zheng et al. 2018). To respond to the rapid spread of airborne diseases, wide-swath sensors with high revisit frequencies are combined with multi-temporal analysis to enable continuous regional monitoring (Zhao et al. 2017). Furthermore, by integrating phenological correction algorithms, the system can distinguish between normal seasonal defoliation and actual disease-induced canopy changes, reducing false positives in deciduous forests and agricultural fields (Zhang et al. 2021). Multimodal approaches that incorporate land surface temperature further increase sensitivity to physiological anomalies, while temporal deep learning models simulate disease progression to support dynamic early-warning systems (Bhattacharya and Chattopadhyay 2013; Bi et al. 2020).

#### Integrated assessment of pest and disease

Unlike diseases, which often manifest as biochemical shifts, chewing or wood-boring pests typically cause physical structural failure. In these cases, spectral information alone is often insufficient. By integrating LiDAR-derived vertical structural data with radiative transfer models, AI can precisely quantify canopy damage and assess pest severity (Lin et al. 2021). In farmland, where pests and diseases frequently co-occur and early-stage samples are scarce, hybrid AI strategies utilize oversampling and rebalancing techniques to mitigate predictive bias and prevent missed detections (Ma et al. 2019). The integration of high-resolution spectral data, sensor fusion, and advanced algorithms has transformed our capacity to monitor pest and disease dynamics across continental scales, providing unprecedented insights for global agricultural management and ecosystem conservation.

## Intelligent predictions of pests and diseases

Effective IPM relies on anticipation, not just reaction. While monitoring identifies current threats, predictive modeling provides the foresight necessary to break the pest cycle before damage occurs. This section examines how computational models operate across short-term tactical decisions for immediate intervention, and long-term strategic planning for seasonal management.

### Real-time early warning frameworks for short-term dynamics

Short-term forecasting shifts agricultural management from rigid, calendar-based spraying to precise, data-driven intervention. By focusing on the micro-level interactions between hosts, pathogens, and their immediate surroundings, these frameworks ensure that countermeasures are applied only when specific biological and economic conditions are met. Biological threats depend on precise microclimate windows to initiate complex physiological processes, such as spore germination, egg hatching, and subsequent host–pathogen interactions. Because broad regional weather reports often miss localized triggers, modern systems utilize wireless sensor networks to capture field data in real time (Kumar et al. 2021; Tripathy et al. 2011). By integrating high-frequency data with deep neural networks, researchers can model fluctuations with high accuracy. This transforms environmental observations into actionable alerts, allowing growers to intervene before a localized outbreak escalates (Fernandes et al. 2017).

Effective IPM, however, requires more than just biological prediction; it necessitates an economic calculation. Interventions are only justified when the potential financial loss from crop damage exceeds the cost of the control measure itself. While traditional models relied on static thresholds, advanced decision-support systems now employ dynamic models that account for fluctuating market prices, expected yields, and real-time crop growth stages (Rincon et al. 2023). Furthermore, these models are increasingly sensitive to the broader ecological context. By incorporating the migration patterns of beneficial insects into the decision-making process, these systems help avoid unnecessary chemical applications that might otherwise disrupt natural pest control (Keasar et al. 2023). This integrated approach ensures that short-term management is both economically efficient and ecologically sustainable.

### Seasonal and climate-driven forecasting for long-term projections

Strategic forecasting extends the horizon from days to months, enabling growers to optimize resource allocation and adjust planting schedules. Unlike short-term models that react to local weather fluctuations, long-term prediction focuses on macro-scale climate patterns and their interaction with pest phenology.

Global warming is disrupting the delicate synchronization between pests and their hosts. Warmer winters increase survival rates, while hotter springs accelerate insect life cycles, creating phenological mismatches. To anticipate these shifts, predictive architectures must move beyond historical baselines and integrate long-term climatic trajectories to capture these evolving dynamics. Recurrent neural networks, such as long short-term memory, are utilized to estimate seasonal development timelines (Atef et al. 2021). Next-generation models further incorporate biological factors to improve the pest establishment predictions in novel territories (Ma and Ma 2022). For highly mobile threats, such as migratory insects, spatial and temporal graph networks map the complex relationships between shifting wind patterns, extreme weather, and geographical features to forecast migration routes and population density (Zhang et al. 2023a). At a broader scale, these frameworks couple global climate projections with localized algorithms to quantify seasonal population pressure across extensive agricultural zones. By leveraging historical climatic anomalies, data-driven networks establish probabilistic thresholds for specific biological threats, enabling growers to anticipate high-risk years and adjust crop varieties accordingly (Taylor et al. 2021).

Furthermore, multidimensional climatic extremes that act as biological triggers or bottlenecks. Heatwaves can shorten development times and relax overwintering barriers, while droughts and unseasonal storms create sudden ecological shocks that conventional linear models fail to process (Deutsch et al. 2018; Monteleone et al. 2023). Recent modeling approaches have been developed to predict dynamics under these extreme conditions, defining new monitoring corridors as pest ranges shift (Hasan et al. 2026). While these systems successfully map climate trends, the ability to dynamically adjust management strategies during unprecedented extreme events remains a critical direction for future research.

## Precision control and intelligent agricultural machinery

**Fig. 4:**
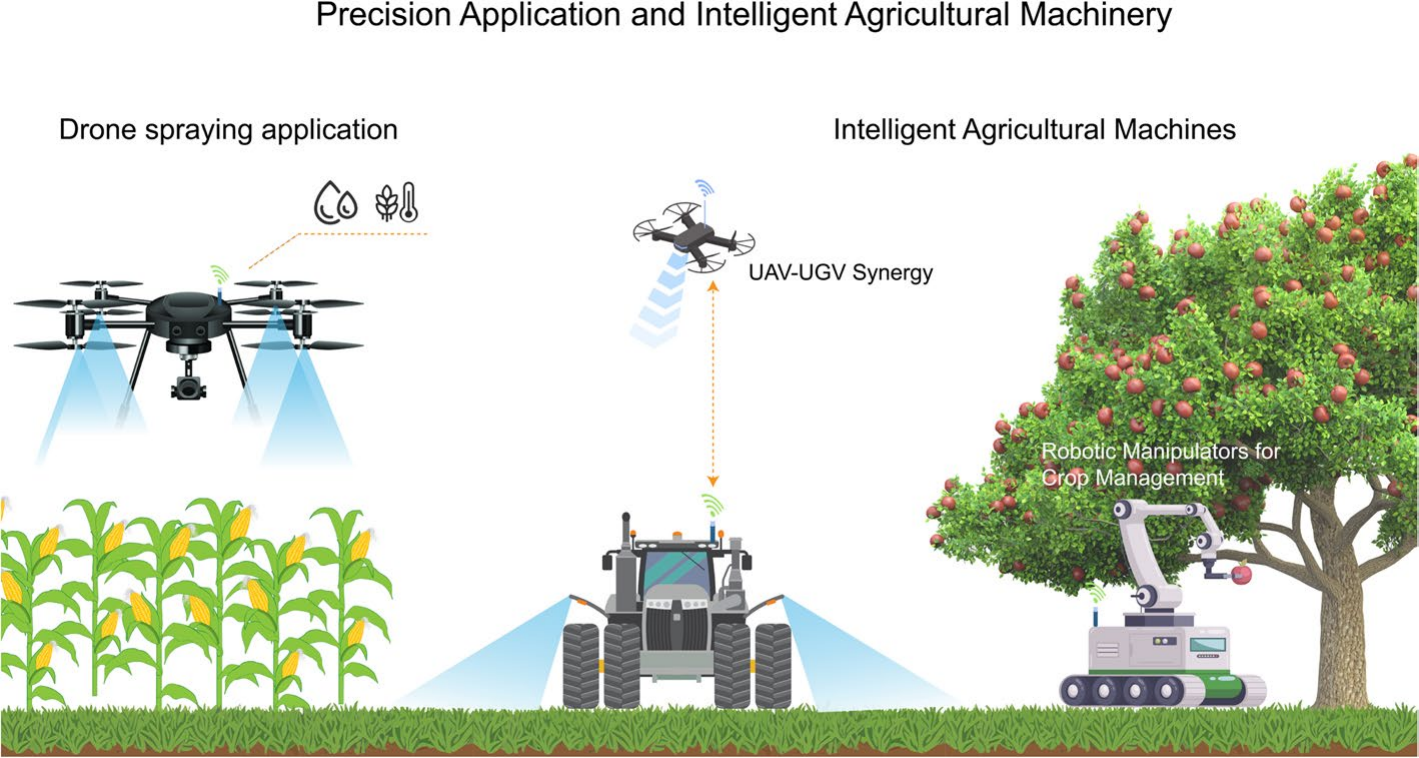
Precision control and intelligent agricultural machinery application

Precision pest management relies on targeted physical or chemical intervention. AI transforms traditional agricultural machinery into intelligent execution systems, deploying algorithms to optimize chemical use and mechanical actions (Fig. 4).

Modern agricultural UAVs have evolved into sophisticated execution platforms that integrate high-resolution sensing with precision spraying mechanisms. Guided by GPS and onboard perception systems, these autonomous drones execute optimized flight trajectories to deliver targeted chemical treatments with high accuracy (Garre and Harish 2018; Radoglou-Grammatikis et al. 2020). By operating entirely above the canopy, UAVs eliminate soil compaction and mechanical crop damage (Ay and İnce 2015). This non-contact approach significantly reduces agrochemical waste and energy consumption, leading to higher yields and a smaller environmental footprint.

Ground-based robotic systems complement these aerial platforms in scenarios requiring heavy payloads, long-term endurance, or direct physical contact. Typically built on unmanned ground vehicles (UGVs) and equipped with multi-jointed robotic arms, these systems manage the full lifecycle of physical intervention. At the frontier of intelligent agriculture, autonomous control systems are increasingly linking aerial and ground platforms through multi-agent coordination. The integration enables an adaptive, fully automated management system capable of addressing biological threats across multiple spatial scales simultaneously.

### Achieving efficient low-altitude interventions via AI empowerment

Modern agriculture faces significant challenges from biotic stresses that threaten crop yields and quality. To address this, precision pesticide application has emerged as a vital alternative to traditional methods. Conventional ground-based spraying often causes mechanical damage to crops, while manual application is prone to uneven coverage. Furthermore, standard aerial spraying carries a high risk of chemical drift into adjacent areas (Wang et al. 2020). In contrast, low-altitude drones allow for targeted treatments without physical crop contact, significantly reducing both environmental contamination and collateral damage.

The structural design of these aerial platforms dictates their suitability for different agricultural scales. Fixed-wing models are optimized for large-area coverage and long-distance flight, whereas multirotor systems offer the maneuverability required for localized, high-precision applications. These platforms utilize a range of power configurations to balance endurance and payload capacity according to specific operational needs. Spray mechanisms have seen similar technical advancements, evolving from simple nozzles to sophisticated pneumatic and electrostatic systems. Notably, variable-rate systems can now achieve near-instantaneous adjustments in fluid delivery. By utilizing real-time pump modulation, these systems maintain a stable and precise spray volume even during the rapid speed or altitude changes common in dynamic flight environments (Lian et al. 2019; Wang et al. 2020).

#### Factors affecting application effectiveness

1. Environmental and Aerodynamic Constraints

Wind speed is the primary driver of droplet drift and deposition. High winds can carry chemicals significantly off-target, while extremely calm conditions may lead to uneven pooling due to rotor interference. To counter this, predictive models are used to establish dynamic buffer zones, often requiring mechanical adjustments like increasing droplet size to resist crosswinds (Chen et al. 2020; Wang et al. 2018, 2020). Temperature and humidity further complicate the process. High temperatures accelerate evaporation, reducing the amount of fluid that actually reaches the leaf, while high humidity can lead to liquid runoff if droplets remain on the surface too long (Chen et al. 2022b; Qi et al. 2020). Adjuvants are frequently added to the spray mix to improve leaf retention and mitigate these atmospheric effects (Sobiech et al. 2020).

2) Precision Flight Parameters

Drone altitude, speed, and swath spacing must be tailored to specific crops. Lower altitudes generally enhance droplet penetration (Wang et al. 2018) but may reduce coverage areas. In citrus application scenarios, identifying the optimal intermediate flight elevation is critical to maximize penetration and volumetric coverage (Tang et al. 2018). Flight speed inversely affects deposition density. This mechanism was clearly validated in rice field interventions (Abd. Kharim et al. 2019). Furthermore, the flight path significantly impacts the uniformity of coverage in complex tree architectures (Meng et al. 2020).

3) Nozzle Technology and Droplet Patterns

Different nozzle types produce distinct droplet patterns, each suited for specific applications (Biglia et al. 2022). Current agricultural drones mainly use hollow cone, flat-fan, and centrifugal nozzles. Hollow cone nozzles offer moderate deposition efficiency but poor drift control, making them suitable for full canopy coverage in orchards. Air-induction flat-fan nozzles provide high deposition efficiency and excellent drift control, ideal for precision-targeted applications. Centrifugal nozzles have low deposition efficiency and moderate drift control, typically used for low-volume ultra-fine droplet spraying. Matching nozzle types, flow rate and crop characteristics is essential for improving deposition and efficacy control.

4) Drone Design and Crop Architecture

The physical design of the drone creates different airflow patterns. Multi-rotor systems generate powerful downdrafts that help push droplets into dense crops like rice or vineyards, whereas helicopter-style drones may be better suited for the high-speed requirements of open fields (Wang et al. 2021; Yallappa et al. 2023). Finally, the shape of the crop itself acts as a physical filter. Uniform structures allow for even distribution, while central leader shapes often trap chemicals in the upper layers, leaving the lower canopy under sprayed.

#### AI-powered decision for drone spraying

Modern AI-driven decision systems have revolutionized pesticide application through optimal timing, precise chemical dosing, and intelligent flight planning. By synthesizing sensor data with optimization algorithms, these systems significantly reduce chemical waste while maximizing biological efficacy.

1. Smart Timing for Maximum Impact

The foundation of effective intervention is real-time environmental sensing and predictive modeling. Advanced deep-learning frameworks analyze high-resolution aerial imagery to generate spatial maps of weed or pest density. This allows the system to transition seamlessly from detection to automated prescription mapping, ensuring that chemicals are applied only when and where they are needed (Guo et al. 2024). Furthermore, by integrating local weather station data with population growth models, these systems can identify the exact microclimatic window that offers the highest treatment success rate.

2) Precision Dosing Technology

Variable-rate spraying represents a major leap in chemical efficiency. Unlike traditional constant-flow systems, AI-enabled drones adjust their dosage in real time based on target characteristics. Sensors measure canopy volume and density, while precision valves adjust the flow rate instantaneously to match the perception layer with the execution layer (Xue et al. 2023). In orchards, enhanced vision algorithms further maximize chemical conservation through targeted tree-by-tree treatment (Wei et al. 2024).

3) Intelligent Flight Planning

Efficient navigation is critical to prevent redundant coverage and environmental pollution. Today’s drones utilize high-fidelity positioning to adhere to complex flight paths with negligible deviation (Zhan et al. 2021). To optimize these routes, researchers deploy advanced algorithms to solve the issue for agricultural fields. These methods determine the most time-efficient waypoints, reducing mission time and energy consumption (Wei et al. 2024). Additionally, swarm coordination allows multiple drones to work in tandem, completing large-scale operations with synchronized precision (Farid et al. 2024). Collectively, these advancements are steering UAV operations toward a fully integrated framework of sensing, decision-making, and execution. Future priorities include building comprehensive parameter databases for diverse crop types and improving the compatibility of eco-friendly organic pesticides with specialized UAV spray systems (Song et al. 2020; Verma et al. 2022).

### Executing sub-canopy operations via ground-based actuation

Ground-based robotic systems, particularly UGVs and specialized manipulators, bridge the gap between sensing and action. These systems must navigate the physical complexities of orchards and fields to perform tasks ranging from autonomous inspection to delicate horticultural tending and automated harvesting.

#### Multi-modal perception and spatial mapping

Effective intervention on the ground requires absolute spatial awareness. To achieve this, AI systems utilize multi-modal sensor fusion, combining stereo vision, 3D point cloud reconstruction, and thermal imaging. This integration allows robots to localize targets with sub-centimeter precision while identifying obstacles and fruit orientation, even when targets are occluded by leaves or share similar colors with the background (Ling et al. 2019).

#### Rule-based precision canopy management

Ground robots have evolved from simple recognition to executing complex maintenance tasks like pruning, thinning, and defoliation. These capabilities rely on rule-based intelligence that translates horticultural expertise into mechanical sequences. Vision-guided robotic arms can now interpret tree architecture to selectively remove branches or thin leaves based on predefined biological criteria, significantly reducing the need for manual labor (Karkee et al. 2014, 2021; Vrochidou et al. 2021).

#### Perception-driven biomechanical efficiency in harvesting

Automated harvesting is the most technically demanding stage of robotic intervention. While early systems struggled with fruit damage and low detachment rates, modern engineering has introduced dual-arm coordination and biomimetic end-effectors. Soft robotic grippers, equipped with real-time force feedback, can now adapt to various fruit geometries to ensure a secure grasp without bruising the delicate produce (Lehnert et al. 2020; Ninatanta et al. 2024). Despite these improvements, overall cycle times remain comparable to earlier systems, indicating persistent efficiency constraints (Bac et al. 2014). To achieve commercial viability, future systems will likely move toward swarm operating systems that distribute tasks across multiple units and utilize cross-modal learning to further accelerate individual action cycles (Ling et al. 2019).

### Ground-air cooperative operations

The integration of UAVs and UGVs is transforming precision agriculture by merging aerial surveillance with ground-based precision operations. UAVs equipped with advanced sensors generate detailed crop health assessments, using multispectral imagery to monitor variables such as crop density, weed pressure, and nitrogen status. These observations enable real-time detection of nutrient deficiencies and weed infestations across large spatial extents (Pretto et al. 2021). The resulting data streams are transmitted to UGVs, which then execute targeted interventions like precision spraying with high spatial accuracy (Chen et al. 2024a).

The synergy between UAVs and UGVs hinges on a robust sensor fusion architecture, which combines aerial and ground-level scanning to achieve exceptional localization accuracy for targeted management (Pretto et al. 2021). Despite advances in adaptive coordination algorithms, the complexity of in-field environments can hinder timely data transmission between platforms. To mitigate this, integrating edge computing modules allows for onboard processing of multispectral data, reducing the decision cycle latency. Machine learning approaches, particularly lightweight neural networks, have demonstrated robust performance in crop-weed classification under variable environmental conditions and unseen fields (Lottes et al. 2018), while semi-supervised methods reduce the need for extensive manual annotation (Lottes and Stachniss 2017). Furthermore, to enable continuous day-night operation, additional sensing strategies such as polarized illumination facilitate reliable nocturnal imaging and improve disease detection performance (Sarkar et al. 2016). However, system recalibration remains time-consuming when switching between different crops, autonomous obstacle response still requires human oversight, and electromagnetic interference from farm equipment continues to affect communication reliability (Huang et al. 2020).

## Challenges in invasive species

Invasive organisms often outcompete native species, posing a severe threat to biodiversity and ecosystem services. Unlike endemic pests, biological invasions expose the limitations of standard agricultural AI across data availability, decision-making logic, and spreading drivers. Traditional predictive models rely on decades of local observations to understand population dynamics. However, newly introduced species exist in a state of spatial disequilibrium. Predicting their trajectory requires AI to integrate multi-source data, linking the species’ physiological traits from its native range with global climate networks to simulate how it might behave in a new environment. Standard pest management is based on coexistence, using economic thresholds to balance crop loss against control costs. This logic fails with invasive species. Without natural predators, these populations grow exponentially and trigger systemic losses that extend far beyond simple yield reductions, damaging broader ecological functions (Khan et al. 2025). Consequently, AI decision systems prioritize early eradication over threshold-based suppression. The drivers of biological invasions differ entirely from those of local pests. While endemic pest dynamics are influenced by field-level microclimates, invasions are propelled by macro-scale forces. Monitoring these threats requires large-scale risk modeling rather than simple field scanning.

### Distribution and spread pathways

#### The dual dilemma of target sparsity and annotation scarcity

Monitoring invasive plants presents two fundamental hurdles: detecting small, scattered individuals within complex natural habitats and overcoming the severe lack of labeled training data for model development. Recent advancements in both sensing technology and analytical methodologies have begun to address these constraints, moving beyond the limitations of traditional manual surveys.

To identify invasive species across diverse ecosystems, researchers are utilizing a hierarchy of imaging technologies. Tailored vegetation indices using specific light bands, such as red-edge and short-wave infrared, enable the mapping of large-scale invasions in coastal and forest environments (Tian et al. 2020). This is complemented by ultra-high-resolution sensors on UAVs, which provide centimeter-level multispectral imagery. Such precision allows for the differentiation of invasive grasses from native vegetation, even in vulnerable high-altitude regions where cloud cover often obstructs satellites (Dao et al. 2021). In dense tropical or temperate forests, fusing optical data with laser scanning (LiDAR) or synthetic aperture radar helps penetrate canopy occlusion to find hidden understory threats (Dai et al. 2020; Xing et al. 2024; Zhao et al. 2022). Despite these hardware gains, certain growth forms remain difficult to track due to their small size or rapid growth cycles. Accuracy is significantly improved by timing observations to match key phenological stages, such as peak flowering, when an invasive species’ spectral signature is most distinct from its surroundings (Kattenborn et al. 2019; Kopeć et al. 2023).

Because acquiring high-quality annotated data is often prohibitively expensive during a new invasion, AI architectures are shifting toward data-efficient learning strategies. Lightweight neural networks have demonstrated the ability to process high-resolution drone imagery effectively using only a minimal number of training samples, often outperforming traditional statistical methods (Chen et al. 2024b; Xing et al. 2024). In aquatic and complex environments, hybrid systems use active learning to reduce the need for manual labeling. By iteratively selecting only the most informative samples for review, these models achieve high accuracy with a fraction of the traditional data requirements (Chowdhury et al. 2024). Collectively, these advancements enhance the scalability of invasion monitoring. While improved sensors provide the necessary observational detail, data-efficient algorithms enable robust detection even when information is scarce. They provide a faster, more scalable framework for ecological conservation than traditional field-based methods alone.

#### Dispersal pathways and driving mechanisms

Unlike the gradual spread of local pests, invasive species often exhibit non-linear jump-dispersal patterns. Identifying and severing these transmission pathways requires a dual analysis of the shifting ecological zones reshaped by climate change and the long-distance vectors provided by global trade and logistics networks.

Climate anomalies play a critical role in opening new habitats. Distribution models have successfully projected the northward expansion of various invasive insects into previously unreachable regions as ecological niches shift (Godefroid et al. 2020; Stoeckli et al. 2020). It is noteworthy that invasive species often exhibit the distinct biological characteristic of limited genetic diversity yet highly robust phenotypic adaptability (Castillo et al. 2018; Gruntman et al. 2020). While most current monitoring systems focus on macro-geographic expansion, incorporating these adaptive trait responses into predictive models is essential for more accurate forecasts of invasion dynamics.

The rapid, long-distance proliferation of biological invasions is fundamentally propelled by global trade and modern transportation corridors. Spatial models and graph theory confirm that major highways and international shipping routes serve as the primary vectors for these jumps (Carrasco et al. 2010; Prasad et al. 2010). By coupling diffusion equations with graph theory, researchers model long-distance dispersal trajectories and pinpoint high-risk invasion centers, allowing for more targeted interception strategies (Cini et al. 2019; Estay et al. 2023). In contrast to human-mediated spread, natural landscape features, such as mountain ranges and river systems, remain the most effective barriers to expansion (Wan and Yang 2016). These features present topographic and climatic limitations that can confine invasive populations to localized zones. Effective future management must therefore find a balance between regulating high-risk anthropogenic corridors while capitalizing on natural geographical barriers to contain spread.

### Invasive species biomass assessment and decision support

Quantifying the biomass of invasive species is essential for assessing both ecological degradation and economic damage. In aquatic systems, for instance, the rapid accumulation of surface biomass is a primary driver of deep-water hypoxia, as dense coverage correlates directly with increased biochemical oxygen demand (Garcés-Gómez et al. 2024; Tang et al. 2022). Beyond simple coverage, understanding how invasive plants allocate biomass, reveals the competitive strategies they use to suppress native flora (Tang et al. 2022). Such findings highlight the importance of resolving biomass components to better understand ecological dominance and competitive strategies.

Furthermore, accurate spatial mapping of biomass is also a prerequisite for developing realistic eradication budgets and allocating intervention resources. High-biomass zones often function as invasion hubs that require prioritized mechanical or chemical control. To manage these interventions across difficult or inaccessible terrain, decision-makers increasingly deploy AI-assisted remote sensing. UAV-based computer vision can accurately estimate plant density to determine the precise volume of conventional herbicide required for a specific area (Huang et al. 2024). Meanwhile, AI-driven algorithms can decode the internal biosynthesis of plant metabolites, facilitating the development of sustainable biological control alternatives (Liu et al. 2026). At a landscape scale, advanced deep learning architectures, such as those combining generative models with cross-feedback frameworks, can extrapolate limited field observations into high-precision biomass maps (Chen et al. 2022a).

Unlike the routine costs associated with managing endemic pests, absolute eradication requires a significantly higher financial investment. By converting remote sensing pixels into precise biomass estimates, these systems provide the intelligent decision support necessary to implement costly eradication protocols with strict budgetary precision. The data-driven approach ensures that limited resources are directed where they will have the greatest impact on protecting biodiversity.

## Discussion and conclusion

### Current predictive lags and inherent theoretical limitations

Research interest in applying artificial intelligence to IPM has surged, with post-2010 breakthroughs solidifying deep learning and UAV systems as main architectures for automated diagnostics and execution. The pronounced phenomenon, where predictive modeling significantly lags behind perception in both publication volume and technical maturity, arises from fundamental task disparities rather than mere developmental oversight. Unlike visual identification, which entails static, closed-set spatial mapping, forecasting is a non-stationary time-series challenge. It demands the nonlinear coupling of high-quality, long-term meteorological and epidemiological data, making it highly susceptible to error accumulation from environmental perturbations. This developmental asymmetry precisely reflects the field’s transition toward complex cyber-physical systems, demonstrating disruptive potential in streamlining a comprehensive perception-to-control chain for both endemic pests and global biological invasions.

Despite distinct trajectories in visual perception and time-series forecasting, agricultural AI faces three inherent theoretical limitations. First, complex models trade interpretability for predictive accuracy, obscuring the agronomic rationale behind recommendations (Khwidzhilli and Ijatuyi 2026; Ryo 2022). Moreover, lacking mechanistic causality, models relying on statistical correlations exhibit profound vulnerability to environmental disturbances. As climate and biological dynamics reshape ecosystems, historical data-driven models risk generalization failures, potentially triggering unanticipated crop losses when deployed at scale without rigorous calibration and validation (Tzachor et al. 2022). Finally, pervasive representation biases in training datasets fail to capture agroecological complexity. Consequently, deploying opaque, correlational, and biased algorithms severely compromises decision-making reliability during critical phytosanitary crises.

### Deployment barriers and systemic solutions

While the integration of multidimensional sensing and AI has redefined pest and invasion management, translating these models into complex, real-world ecosystems remain a formidable challenge. At the perception level, physical biosensors suffer from degraded signal-to-noise ratios due to wind, drastic illumination shifts, and atmospheric noise, severely compromising edge device accuracy. High-precision models trained in controlled laboratory environments frequently exhibit significant fragility in actual fields. Environmental heterogeneity further leads to a sharp decline in the algorithmic generalization when applied across different crops, pathogens, and climatic regions, a challenge further exacerbated by the need for highly localized control interventions. Moreover, high deployment, computational costs, and the fundamental mismatch between cloud-reliant AI architectures and unstable rural networks severely constrain real-world accessibility, particularly for smallholder farmers.

Surmounting the triad of field deployment barriers, inequitable accessibility, and standardization deficits demands convergent evolution across hardware augmentation, algorithmic adaptability, and macro-architectural design. To mitigate sensor fragility, physical anti-interference enhancements (Cellini et al. 2017) need be coupled with domain adaptation algorithms. At the systemic level, developing “cloud-edge-device” coordination models tailored for resource-constrained environments is imperative, utilizing decentralized edge autonomy to mitigate latency caused by network fluctuations. From a cost–benefit perspective, decision-support frameworks must dynamically balance diagnostic precision with resource allocation to prevent the over-application of costly interventions on low-value crops. Crucially, at the data and policy dimensions, transcending data silos requires establishing open-source, cross-climatic benchmark datasets, while policy-driven initiatives must assimilate scattered agronomic practices into standardized knowledge graphs. This strategic knowledge migration transforms isolated human empiricism into shared machine priors, fundamentally enabling robust, cross-regional autonomous interventions. Finally, ethical AI deployment necessitates stringent regulatory policies and standardized protocols to ensure transparency, fairness, and environmental accountability, ultimately aligning technological progress with broader socio-ecological goals.

The integration of AI with multidimensional sensing marks a transformative era in plant health management, enabling unprecedented precision in detection and intervention. This synergy fostered a closed-loop system where AI does not merely interpret signals but actively directs smart machinery to optimize field conditions in real-time. As climate change and global trade intensify biological threats, these challenges will transcend individual farmland boundaries to impact entire biomes, including forests and wetlands.

## Data Availability

No datasets were generated or analyzed during the current study.
